# Rechallenge After Clozapine-Induced Neutropenia: A 30-Year Case Series From a UK Mental Health Trust

**DOI:** 10.1192/bjo.2026.11946

**Published:** 2026-06-30

**Authors:** Mohamed Ismail, Sumeet Gupta, Liyana Mohamed, Olofunto Segun, Farhad Faridhosseini

**Affiliations:** 1 Humber NHS Trust, Hull, United Kingdom; 2 TEWV NHS Trust, York, United Kingdom; 3 TEWV NHS Trust, Harrogate, United Kingdom; 4 LYPFT NHS Trust, Leeds, United Kingdom; 5 BDCT NHS Trust, Bradford, United Kingdom

## Abstract

**Aims:**

Clozapine is the only evidence-based medication for treatment-resistant schizophrenia and is associated with reductions in relapse, suicide risk, and all-cause mortality.Nevertheless, its use is restricted by concerns about adverse effects, particularly neutropenia and agranulocytosis, which often result in permanent discontinuation under current UK monitoring rules.

Emerging evidence and regulatory changes, particularly in the United States and Europe, suggest that Clozapine re-challenge may be safe in selected patients following careful individualised risk–benefit assessment. However, Clozapine re-challenge remains uncommon in the UK.

**Methods:**

We completed a retrospective case series of patients who underwent Clozapine re-challenge within TEWV NHS Trust.Using CPMS data and clinical records, patients who stopped Clozapine due to neutropenia between 1994 and 2024 were identified. We then checked the data of patients who were re-challenged with Clozapine in detail. Extracted data included demographic characteristics, severity and duration of neutropenic episodes, time to neutropenia following Clozapine initiation, interval between discontinuation and re-challenge, re-challenge strategies (including use of lithium and/or G-CSF) and clinical outcomes.

Successful re-challenge was defined as continued Clozapine treatment without recurrent red blood results requiring permanent discontinuation.

**Results:**

We found that thirteen (46%)out of twenty-eight patientswho discontinued Clozapine due to neutropenia underwent re-challenge.

Eight patients (61.5%) were successfully re-challenged and maintained on Clozapine.

Five re-challenges were unsuccessful and three of which were associated with recurrent severe neutropenia.

No fatalities occurred following Clozapine re-challenge.

Most cases of Clozapine re-challenge were undertaken using Clozapine alone, without adding lithium or G-CSF.

With the exception of two cases, all index neutropenic events occurred beyond the first year of Clozapine treatment, ranging from 17 months to 20 years.

Neither the severity nor the timing of the initial neutropenic episode reliably predicted the rechallenge outcome.

**Conclusion:**

Clozapine re-challenge was successful in a majority of patients, with no deaths observed. Neither severity nor timing of the index neutropenic episode reliably predicted the re-challenge outcome. Therefore,Clozapine re-challenge should be cautiously considered in selected patients, particularly where no effective alternatives exist.

These findings highlight the need for a more flexible and evidence based guidance for Clozapine monitoring and re-challenge.

